# Mass Spectrometric Studies of Reductive Elimination from Si(IV) Anions

**DOI:** 10.1002/chem.202501643

**Published:** 2025-07-24

**Authors:** Pamela Adienes Benzan Lantigua, Mònica Rodríguez, Jana Roithová, Marc‐Etienne Moret

**Affiliations:** ^1^ Organic Chemistry and Catalysis Institute for Sustainable and Circular Chemistry Faculty of Science Utrecht University Universiteitsweg 99 Utrecht 3584CG The Netherlands; ^2^ Department of Spectroscopy and Catalysis Radboud University Nijmegen Heyendaalseweg 135 Nijmegen 6525AJ the Netherlands

**Keywords:** ESI‐MS, main‐group elements, reaction mechanisms, reductive elimination, silicon

## Abstract

Reductive elimination (RE) is one of the most significant obstacles to developing main‐group catalysts for bond coupling processes based on conventional oxidative addition/reductive elimination steps. Here, we report an electrospray ionisation mass spectrometry (ESI‐MS) study demonstrating RE from Si^IV^ hydrosilicates [(tmim)SiH(OAr^R^)]^−^ (tmimH_3_ = tris(3‐methylindol‐2‐yl)methane) generated in solution by oxidative addition of phenols (Ar^R^O─H) to an anionic Si^II^ centre (silanide) incorporated in a bicyclic cage structure ([(tmim)Si]^−^). The activation energy for the reductive elimination linearly decreased with the increasing electron‐withdrawing character of the aryl substituents, suggesting that a negative charge develops in the para position in the rate‐limiting step (RLS). In agreement with experiments, a DFT study supports an ionic mechanism in which the formation of an ion/molecule complex by transient weakening of the Si─O bond is the RLS, as would be intuitively expected from the developing negative charge in the para position. This is a rare example of direct observation of reductive elimination from Si^IV^ to Si^II^ and, to our knowledge, the first involving anionic species.

## Graphical abstract

Reductive elimination of ArO─H (Ar = aromatic residue) bonds from pentacoordinate Si^IV^ is characterized by mass spectrometric methods. A Hammet study of substituent effects combined with DFT calculations supports a stepwise mechanism involving a loosely bound aryloxide/hydrosilane ion‐molecule complex as an intermediate.

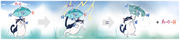

## 1 Introduction

The ability of transition metals (TMs) to couple changes in (formal) oxidation state to the breaking and making of chemical bonds underpins their versatile use in catalysis.^[^ ^1^, ^2^ ^]^ Compounds of earth‐abundant main‐group elements that can mimic elementary reactions such as oxidative addition (OA) and reductive elimination (RE) are emerging as potential environment‐friendly alternatives to TM‐based homogeneous catalysts.^[^ ^3^, ^4^, ^5^, ^6^, ^7^, ^8^, ^9^, ^10^, ^11^, ^12^ ^]^ While many examples of oxidative addition (OA) at low‐valent main‐group centres have been documented, the challenging nature of the reverse reaction hampers the development of productive catalytic cycles.^[^ ^9^, ^13^, ^14^ ^]^ The relative ease of RE reactions in TM complexes relates to the accessibility of their lower oxidation states, which contrasts with several main‐group elements for which the reduced state is thermodynamically uphill.^[^ ^9^ ^]^

**Figure 1:**
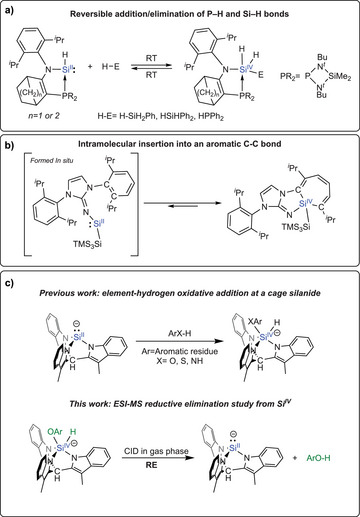
Examples of RE reported in the literature A and B), previous work describing the oxidative addition of polar H─X bonds to a silanide bicyclic cage structure C, top), and the current study showing the induced gas‐phase reductive elimination from the phenolated compounds C, bottom).

Low‐valent silicon compounds, such as neutral silylenes, undergo a variety of OA reactions to form the corresponding Si(IV) compounds. Examples include the activation of small molecules (H_2_, CO_2_, N_2_O),^[^ ^5^, ^8^, ^9^, ^15^, ^16^, ^17^, ^18^ ^]^ polar element‐hydrogen bonds^[^ ^19^ ^]^ including N─H,^[^ ^5^, ^20^ ^]^ O─H,^[^ ^21^ ^]^ S─H,^[^ ^20^ ^]^ as well as various other functional groups ^[^ ^7^, ^22^ ^]^ such as ketones,^[^ ^23^, ^24^ ^]^ aldehydes,^[^ ^22^ ^]^ alkenes, and alkynes.^[^ ^25^ ^]^ The less studied anionic Si^II^ compounds (silanides, [R_3_Si^−^]) have recently been shown to display biphilic reactivity akin to the neutral silylene analogues by the group of Hoge.^[^ ^26^, ^27^ ^]^ Work from Cowley,^[^ ^28^ ^]^ showed a promising catalytic application of the (Me_3_Si)_3_Si^−^ anion. Recently, we showed that a silanide incorporated bicyclic cage structure ([(tmim)Si]^−^, tmimH_3_ = tris(3‐methylindol‐2‐yl)methane) can undergo facile oxidative activation of polar element‐hydrogen bonds (ArX─H, Ar = aromatic residue, X = O, S, NH) to generate the corresponding hydrosilicates (Figure 1c, top).^[^ ^29^ ^]^

While examples of RE involving heavier germanium and tin analogues have been known for some time,^[^ ^13^, ^30^, ^31^, ^32^ ^]^ examples of well‐defined RE at silicon are very scarce.^[^ ^33^, ^34^, ^35^ ^]^ Kato et al. have shown that the oxidative addition of Si─H and P─H σ‐bonds to their phosphine‐stabilized Si^II^ centers centers was reversible at room temperature, demonstrating that RE can take place at silicon (Figure 1a).^[^ ^36^ ^]^ Moreover, Inoue et al. described an acyclic Iminosilylsilylene that undergoes reversible intramolecular insertion into an aromatic C─C bond to form a silepin, from which reductive elimination to Si(II) is largely driven by rearomatization (Figure 1b).^[^ ^37^ ^]^ This work was later extended to the reversible, intermolecular insertion of an iminosilylene into aromatic rings.^[^ ^38^, ^39^ ^]^

To further assess the viability of catalytic reactions involving the Si^II^/Si^IV^ couple, we set out to gather mechanistic information on the RE from Si^IV^ hydrosilicates (Figure 1c, bottom). Taking advantage of their negative ionic nature, electrospray ionisation mass spectrometry (ESI‐MS) provides an opportunity to study the intrinsic reactivity of these Si^IV^ species in the gas phase. Indeed, the sensitivity of ESI‐MS makes it possible to detect elusive intermediates, even though they are present with low abundance in the solution. When the compounds are transferred to the gas phase, they can be isolated from other reactants, allowing for an in‐depth study of their reactivity. Given that the RE is a unimolecular process, it can be induced via Collision‐Induced Dissociation (CID) using a non‐reactive collision gas, which will elevate the internal energy of the desired ion. This approach will promote the RE of the targeted ions if it represents the lowest‐energy fragmentation pathway of the ions under study.

In this work, we report an ESI‐MS study of the RE of Si^IV^ hydrosilicates [K(18‐crown‐6)] [(tmim)SiH(OAr)] using collision‐induced dissociation (CID) experiments (Figure 1, bottom). These experiments demonstrate that RE can be effectively induced in the gas phase for these Si^IV^ compounds and reveal how the electronic properties of the eliminated fragment impact the RE pathway. Furthermore, Density Functional Theory (DFT) supports the observed experimental findings, providing insights into substituent effects and supporting an ionic mechanism governing the RE step.

## 2 Results and Discussion

### 2.1 Detection and Fragmentation of **2^Me^**

We have previously reported the isolation and characterization of cage silanide **1**, and showed that this structure could undergo oxidative addition with various phenol derivatives to form the hydrosilicate [K(18‐crown‐6)] [(tmim)SiH(OAr)] (Figure 1, bottom). These phenol derivatives included substituents such as methyl, methoxide, chlorine, and nitrile groups.^[^ ^29^ ^]^

**Figure 2:**
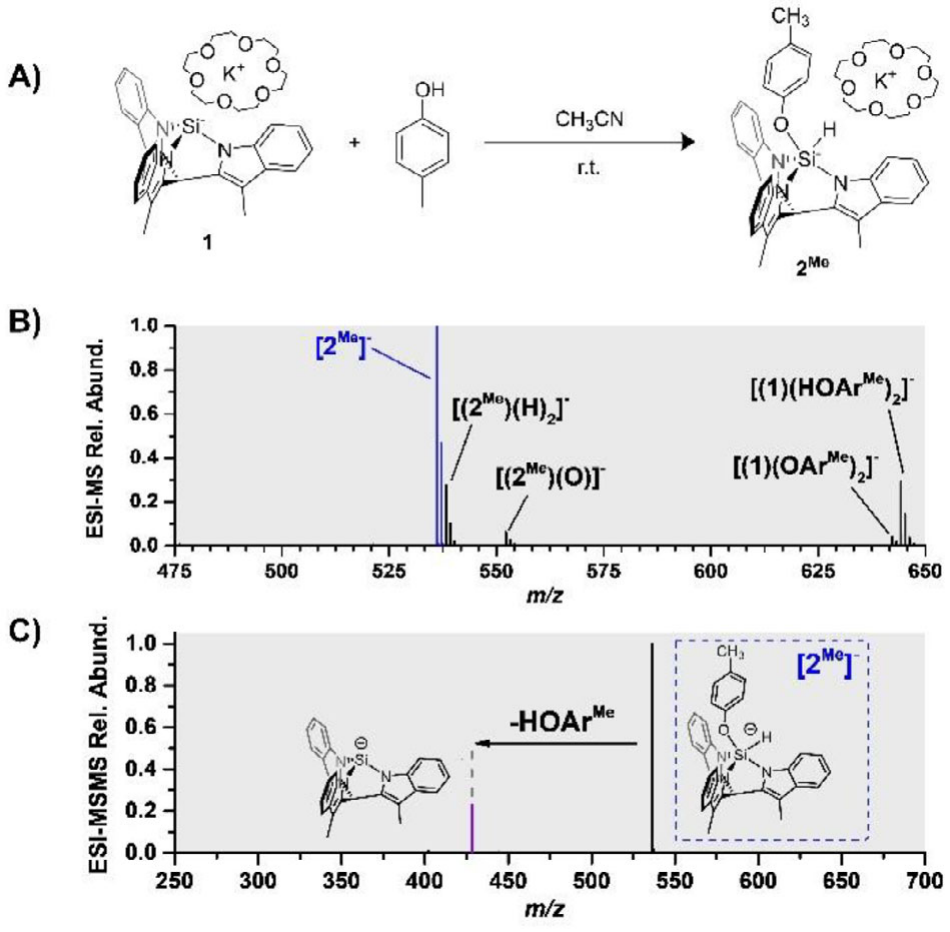
A) Scheme of the oxidative addition of p‐cresol to **1** B) CSI‐MS spectrum of **2^Me^**; C) Collision‐induced dissociation (CID) spectrum of mass‐selected **[2^Me^]^−^** at the collision energy *E*_lab_ = 16 eV (laboratory frame).

In this study, we analysed one of these derivatives, **2^Me^** (Figure 2A), by cold‐spray ionisation in negative mode (CSI(‐)‐MS) at −40 °C. The CSI(‐)‐MS analysis of a **2^Me^** solution in MeCN revealed the most intense signal at *m/z* = 536.21, corresponding to **[2^Me^]^−^**, indicating the negatively charged silanide upon the loss of the crown ether cation (Figure 2B). Additionally, minor decomposition species such as **[(2^Me^)(H)_2_]^−^** or **[(2^Me^)(O)]^−^** were observed too. Cold‐spray ionisation was used over electrospray ionisation to minimise the decomposition of **2^Me^** to **[(2^Me^)(H)_2_]^−^** in the source. The **[(2^Me^)(H)_2_]^−^** ions most likely correspond to the adduct of Ar^Me^OH with the Si(II)‐hydride with singly protonated ligand (see Supporting Information Section S2.1 for ESI‐MS analysis and assignment of the decomposition products).

CID of mass‐selected **[2^Me^]^−^** led to the loss of a HOAr^Me^ molecule and recovery of **[1]** (Figure 2C). In comparison, CID of the decomposition products **[(2^Me^)(H)_2_]^−^** and **[(2^Me^)(O)]** also reveals the Ar^Me^OH elimination, but requires lower collision energies, indicative of a simple ligand decoordination process. On the contrary, for **[2^Me^]^−^**, the phenol loss occurs at higher collision energies, demonstrating the necessity of higher energy input to overcome the reductive elimination energy barrier.

### 2.2 Electronic Modification of **2^R^** to Modulate the Reductive Elimination Step

**Figure 3:**
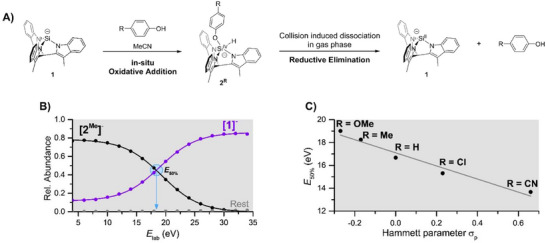
A) Reaction scheme with an oxidative addition of para‐substituted phenols with **1** in solution, followed by a reductive elimination promoted in the gas phase. B) Energy‐resolved CID curves for the reductive elimination of **[2^Me^]^−^** to **[1]^−^**. The intersection point (*E*_50%_) between the two curves is used as a qualitative indicator of the relative dissociation energy. C) Hammett plot representing the *E*_50%_ values of the different substituted phenols versus the Hammett parameter for substituents in para position.

Having proven the feasibility of studying RE from **2^Me^** in the gas phase, we explored the effect of electronic modifications of the phenol molecule on the RE step. Due to the high sensitivity of these compounds, oxidative addition products were generated in situ, by mixing precursor **1** with various para‐substituted phenols at −40 °C under strictly inert conditions just seconds before the injection of the sample into the CSI‐MS (Figure 3A).

A peak corresponding to the desired oxidation product **[2^R^]^−^** (R = OMe, H, Cl, CN) appeared immediately after the addition of the para‐substituted phenols to the solution of **1** (See Supporting Information Section S2.3 and Figure S5). CID fragmentation of the mass‐selected ions showed the expected loss of HOPh^R^ as the main fragmentation pathway. To compare how the electronic properties affect the energy demand on the reductive elimination pathway, energy‐resolved collision‐induced dissociation for each species was performed. Note that the energy results can only be considered as qualitative values rather than quantitative bond dissociation energy values. Our experimental setup did not allow us to make energy‐resolved single‐collision experiments, allowing quantitative modeling of bond dissociation energies.^[^ ^40^, ^41^ ^]^ Instead, we followed a consistent protocol for the energy resolved CIDs, monitoring the depletion of the parent ion and the growth of **[1]^−^** at a constant collision‐gas pressure. As a measure of the dissociation energy, we used the energy, where 50% of the parent ions converted to **[1]^−^** (*E*_50%_, Figure 3B). Plotting the *E*_50%_ values of **[2^R^]^−^** against the corresponding Hammett parameters reveals a linear correlation where electron‐withdrawing groups facilitate reductive elimination at lower collision energy (Figure 3C and Figure S6).

**Figure 4:**
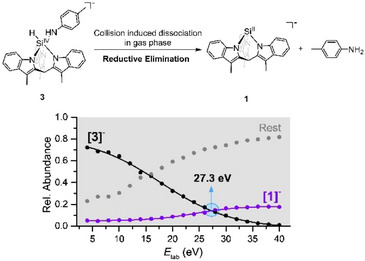
Scheme of the reductive elimination step in the gas phase (top). Energy‐resolved CID curves for the reductive elimination of **[3]^−^** to **[1]** (bottom).

Next, we studied reductive elimination from in situ generated silicate **[3]^−^**, which contained a deprotonated aniline group instead of a phenolate (Figure 4). The **[3]^−^** complexes are more sensitive than the **[2^R^]^−^** phenol analogues, and their abundance in the CSI‐MS spectrum is very low. Nevertheless, thanks to the high sensitivity of mass spectrometry, it was possible to mass‐select the ions and perform the energy‐resolved CID (Figure 4, Supporting Information Section S2.4; Figures S8 and S9). The **[3]^−^** ions showed a low propensity of RE and large amounts of other decomposition products, corresponding to the NH_2_‐bound silanide complex as the most abundant by‐product, together with ligand fragmentation, CH_3_‐C_6_H_4_‐NH**^−^**, and other unidentified fragments (Figure S9). The relative energy required for the RE for **[3]^−^** significantly exceeds that observed for phenol‐based compounds, thereby supporting the hypothesis that increasing acidity aids the RE process.

### 2.3 Computational Study: RE Process

**Figure 5:**
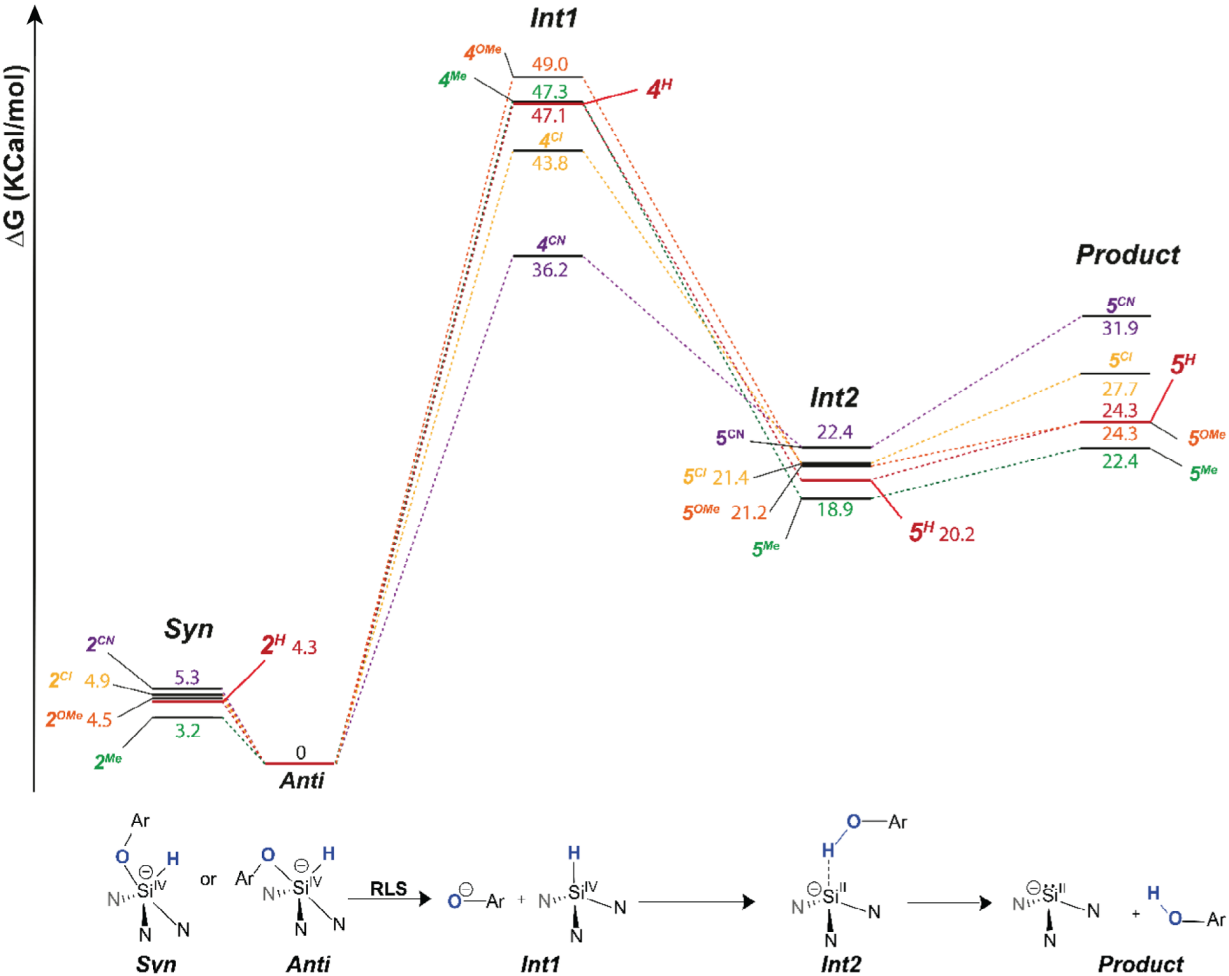
Computed free energies for the proposed ionic mechanism for reductive eliminationfrom **2^R^** (R = H, Me, OMe, Cl, CN) at the B3LYP‐GD3BJ/6–311++G(d,p)//6–31G(d,p) level of theory (Top) and scheme of the reaction mechanism (bottom).

To gain more insight into the results obtained by the CSI‐MS study, the reductive elimination (RE) process for the phenol‐based hydrosilicates was studied using Density Functional Theory (DFT). The lowest energy hydrosilicate conformer (**anti**) was used as the starting point. The calculations (Figure 5) suggest a rebound‐like ionic mechanism, which is the microscopic reverse of that computed for the oxidative addition of phenols to cage silanides.^[^ ^29^ ^]^ First, the ArO^−^ anion group detaches from the silicon center to form a loosely bound ion‐molecule complex (**int1**). Then, the phenolate abstracts a proton from the neutral silane to form a hydrogen‐bonded phenol/silanide complex (**int2**), from which the phenol is released as a neutral fragment. Based on this ionic mechanism, it may appear surprising at first sight that CID fragmentation of the starting hydrosilicate does not produce a large amount of free phenolate from the dissociation of the ion‐molecule complex **Int1**. This can be explained by the fact that both the heterolytic Si─O cleavage and the subsequent Si─H deprotonation are devoid of electronic barriers.^[^ ^29^ ^]^ Hence, even though it is only held together by weak ion‐molecule interactions, the ion‐molecule complex lives long enough to collapse back to either starting materials or products without dissociating. An alternative explanation for the observation of clean reductive elimination could be a different, concerted mechanism, but such mechanisms are disfavoured by computations (See Supporting Information). In addition, an alternative mechanism involving initial cleavage of an Si─N bond instead of the strong Si─O bond was computed to have a significantly higher energy barrier (See Supporting Information). An additional way to distinguish the proposed ionic mechanism from an alternative concerted one relies on the experimental observation that electron‐withdrawing substituents accelerate the reductive elimination process (vide supra).

**Figure 6:**
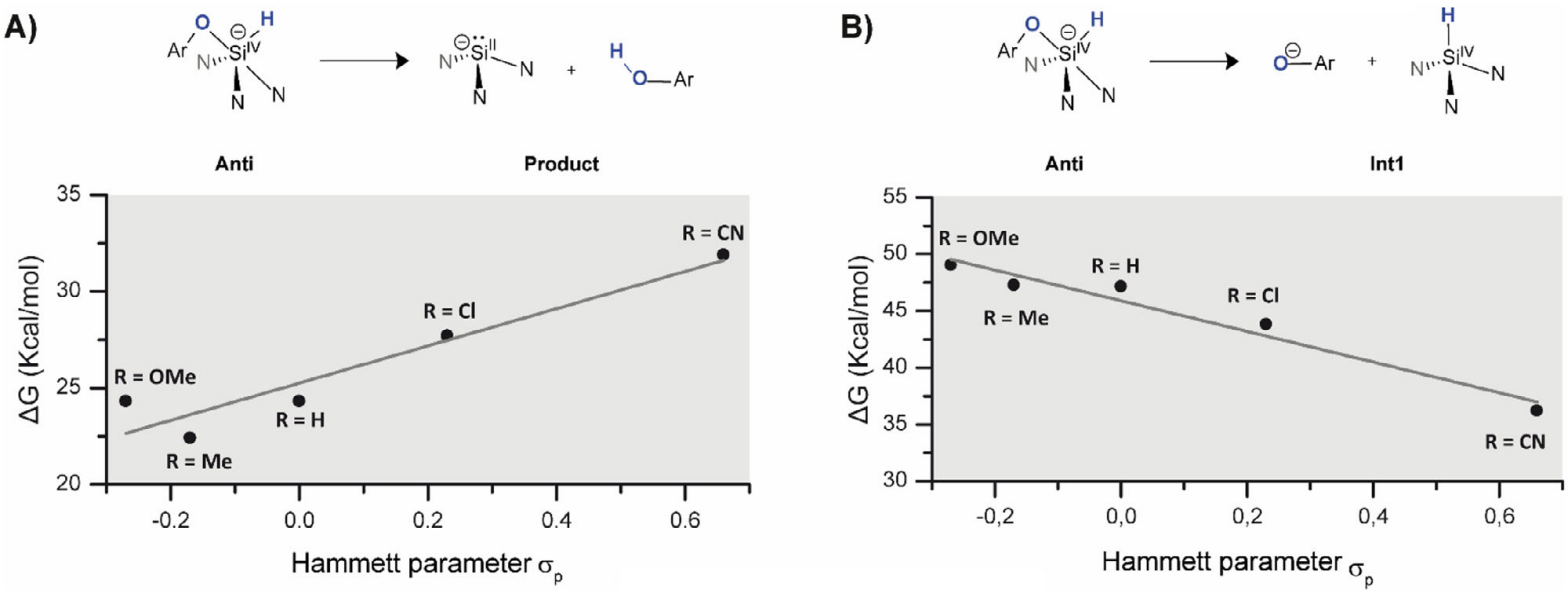
Hammett plot representing the energy of the reaction toward the **product** A) and **int1** B) for the different substituted phenols versus the Hammett parameter for substituents in the para position.

According to the Bell‐Evans‐Polanyi principle,^[^ ^42^ ^]^ one would expect substituent effects on the rate of reaction to parallel those on the overall free energy of reaction for a hypothetical concerted reaction. However, plotting the energy values of the **Product** against the corresponding Hammett parameters (Figure 6a) reveals that the overall reductive elimination reaction is thermodynamically favoured by electron‐donating substituents. This discrepancy confirms the presence of a rate‐limiting step (RLS) with different electronic requirements prior to the dissociation of the products, at odds with a hypothetical concerted mechanism. In the proposed ionic mechanism in Figure 5, the RLS is the formation of the ion/molecule complex **int1** for all investigated substituents. Plotting the energy values calculated for the ion/molecule complex **int1** against the corresponding Hammett parameters (Figure 6b) confirms that electron‐withdrawing substituents facilitate the formation of **int1,** as would be intuitively expected from the developing negative charge in the *para* position. This is in good agreement with the CSI‐MS experiments and lends credence to the proposed ionic mechanism. In a related study, Bielawski and coworkers have used electronic effects on the oxidative addition of substituted anilines to different carbenes to distinguish between electrophilic and nucleophilic pathways.^[^ ^43^ ^]^

## 3 Conclusions

In summary, we have demonstrated that reductive elimination (RE) from Si^IV^ hydrosilicate compounds can be effectively studied in the gas phase. Accordingly, we elucidated the Si^IV^/Si^II^ reaction mechanism using a combination of experimental and theoretical methods. Varying the electronic properties of Si^IV^ aryloxo hydrosilicate complexes uncovered an accelerating effect of the electron‐withdrawing substituents at the aryloxo (phenolate) on the RE step, which is consistent with an ionic mechanism. DFT calculations support initial heterolytic cleavage of the Si─O bond to form a phenolate/silane ion‐molecule complex; the phenolate then abstracts a proton from the Si^IV^‐H core to form phenol and the product Si^II^ anion. These results offer key insights into the electronic factors governing reductive elimination at Si^IV^ and pave the way toward further exploration of main‐group complexes as catalysts.

## Supporting Information

The authors have cited additional references within the Supporting Information.^[^ ^44^ ^]^

## Conflict of Interest

The authors declare no conflict of interest.

## Supporting information



Supporting Information

## Data Availability

The data that support the findings of this study are available in the supplementary material of this article.
