# Supplementary material for: Mass Spectrometric Studies of Reductive Elimination from Si(IV) Anions
Source: Chemistry. 2025 Jul 24;31(47):e01643. doi: 10.1002/chem.202501643 (PMC12376247; doi:10.1002/chem.202501643)
Supplement: Supplementary file 1 — Supporting Information [file CHEM-31-e01643-s001.pdf]

Supporting Information
©Wiley-VCH 2021
69451 Weinheim, Germany

# Mass Spectrometric Studies of Reductive Elimination from Si(IV) Compounds

Pamela Adienes Benzan Lantigua, Mònica Rodríguez , Jana Roithová* and Marc-Etienne Moret*

**DOI: 10.1002/anie.2021XXXXX**

## Table of Contents

1. Experimental Section ..... 3
1.1. General Information ..... 3
1.2. Physical Methods ..... 3
1.2.1. Electrospray mass spectrometry experiments. ..... 3
2. Additional experiments ..... 4
2.1. Optimization of the ESI-MS measurement of $2^{Me}$ ..... 4
2.2. Collision-induced dissociation of $2^{Me}$ and decomposition species ..... 5
2.3. Electronic modification of $2^{R}$ to modulate the reductive elimination step ..... 7
2.4. ESI-MS of 3 ..... 9
3. DFT studies ..... 10
3.1. Computational methods ..... 10
3.2. Study of the RE mechanism ..... 10
3.3. Energies ..... 12
4. References ..... 14
Author Contributions ..... 14

# 1. Experimental Section

## 1.1. General Information

All reactions involving air-sensitive compounds were conducted under an $N_2$ atmosphere by using standard glovebox or Schlenk techniques. Acetonitrile, *n*-hexane and $Et_2O$ were dried with an MBRAUN MB SPS-79 system. THF was distilled from benzophenone/Na. All solvents were degassed by bubbling with $N_2$ and stored over molecular sieves in a glovebox. Unless otherwise mentioned, all chemicals used in this work, were commercial products of the highest available purity and were further purified by standard methods, if necessary. Deuterated MeCN and THF were degassed by three freeze-pump-thaw cycles and stored over molecular sieves in a glovebox, except for acetonitrile which was stored without sieves. Compound **1**, his precursors (Idipp·$SiCl_2$ and (tmim)$K_3$) and **2**$^{\mathbf{Me}}$ were prepared according to the reported procedures.[1,2]

## 1.2. Physical Methods

All NMR measurements were performed on a Varian VNMRS400 or Varian MRF400 spectrometer. The $^{1}H$ and $^{29}Si$ chemical shifts are reported relative to TMS with the residual solvent signal as internal standard when possible. All NMR experiments involving air-sensitive compounds were conducted in J-Young NMR tubes under an $N_2$ atmosphere.

### 1.2.1. Electrospray mass spectrometry experiments.

The experiments were performed in a time-of-flight mass spectrometer (timsTOF, Bruker Daltonics, Bremen, Germany) operating in negative mode. Ions were generated by coldspray ionization using the Cryospray unit with the following settings (Capillary voltage -3 kV, sprayer pressure 0.088 MPa and drying gas flow 2 L.min$^{-1}$). To minimize the decomposition of reactive intermediates, the sprayer and drying gas were both held at -40°C. Typical ion transfer voltages were quadrupole ion energy= -4 eV and collision energy = -4 eV. The collision gas inflow was set at 64%.
The solution samples of **2**$^{\mathbf{Me}}$ and **3** were prepared inside a glovebox using dry acetonitrile in a Schlenk flask. The solution was taken outside the glovebox, cooled down to -40°C to minimize the decomposition, and injected into the mass spectrometer under argon overpressure.

## 2. Additional experiments

### 2.1. Optimization of the ESI-MS measurement of $2^{Me}$

When an acetonitrile solution of **2Me** was injected in the ESI-MS, the most intense peaks observed correspond to decomposition products, due to the high sensitivity of the compound (Figure S1, top). To try to avoid its decomposition in the source as much as possible, we injected the compound via cold-spray ionization at -40°C and we could observe how the decomposition was diminished, being m/z = 536.21 ($2^{Me}$) the most intense peak observed (Figure S1, bottom).

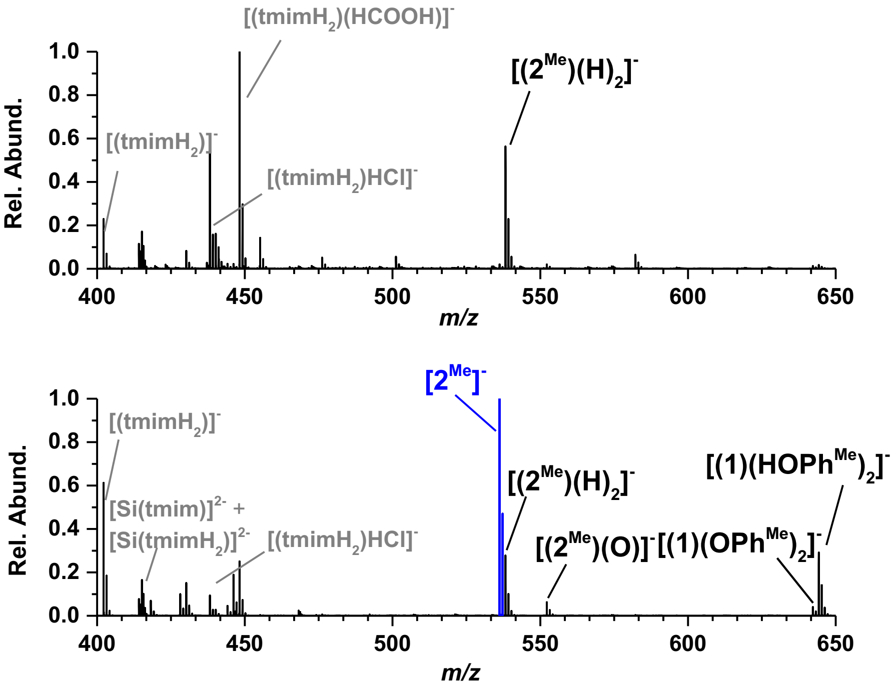


**Figure S1.** ESI-MS spectrum of $\mathbf{2^{Me}}$ where mainly decomposition products can be observed (Top) and using cold-spray ionization at -40°C, where $\mathbf{2^{Me}}$ is the most abundant peak (Bottom).

Nevertheless, relevant decomposition peaks could be observed: the protonation of $2^{Me}$ to generate $Si^{II}$ complex **($[(2^{Me})(H)^2]^-$**, m/z = 538.23), the oxidation of $\mathbf{2^{Me}}$ (**$[2^{Me})(O)]^-$,** m/z = 552.21), and the coordination to **1** of two phenolate groups (**$[(1)(OPh^{Me})_2]^-$**, m/z= 642.26) or two phenol molecules (**$[(1)(HOPh^{Me})_2]^-$,** m/z= 644.27) (Figure S2).

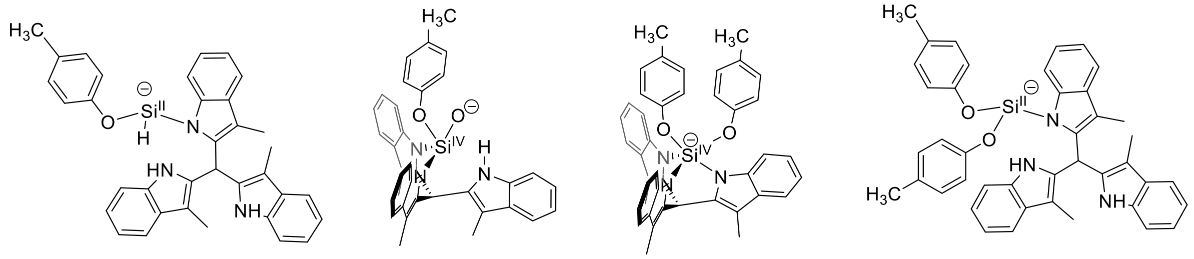

$[(2^{Me})(H)_2]^-$ $[(2^{Me})(O)]^-$ $[(1)(OPh^{Me})_2]^-$ $[(1)(HOPh^{Me})_2]^-$

**Figure S2.** Structures of the decomposition species observed for $\mathbf{2^{Me}}$.

### 2.2. Collision-induced dissociation of $2^{Me}$ and decomposition species

Mass selection of $\mathbf{2^{Me}}$ ion and further fragmentation by collision-induced dissociation (CID) shows the loss of $HOPh^{Me}$ (Figure S3, A). To corroborate if the ion corresponds to the proposed structure for $\mathbf{2^{Me}}$ and the loss comes from a reductive elimination step induced in the gas phase, and not to a simple adduct of phenol with the silanide **1** complex, we performed CIDs of all related decomposition products at the same collision voltage and we compared its fragments and fragmentation intensities. Fragmentation of the ion $\mathbf{[(2^{Me})(H)_2]^-}$ also shows as the main fragmentation ion the loss of $HOPh^{Me}$, together with a small fragmentation path where the complex is completely dissociated, leading to the negatively charged ligand (loss of $SiHOPh^{Me}$). The main difference from $\mathbf{2^{Me}}$ arises from the fragmentation intensity. While $\mathbf{2^{Me}}$ only fragments around 20%, approximately 90% of the parent $\mathbf{[(2^{Me})(H)_2]^-}$ is fragmented towards the loss of $HOPh^{Me}$ (Figure S3, B). These results suggest that while for the latter, the easier fragmentation comes from the dissociation of a coordinated molecule of phenol, for the former, the more difficult fragmentation could arise from the necessity to overcome the barrier for the reductive elimination step. Similarly, for the ion $\mathbf{[(2^{Me})(O)]^-}$, the main fragmentation path comes again with the loss of $HOPh^{Me}$, also fragmenting around 90% of the parent, attributing it to the presence of a phenol molecule coordinated (Figure S3, C).

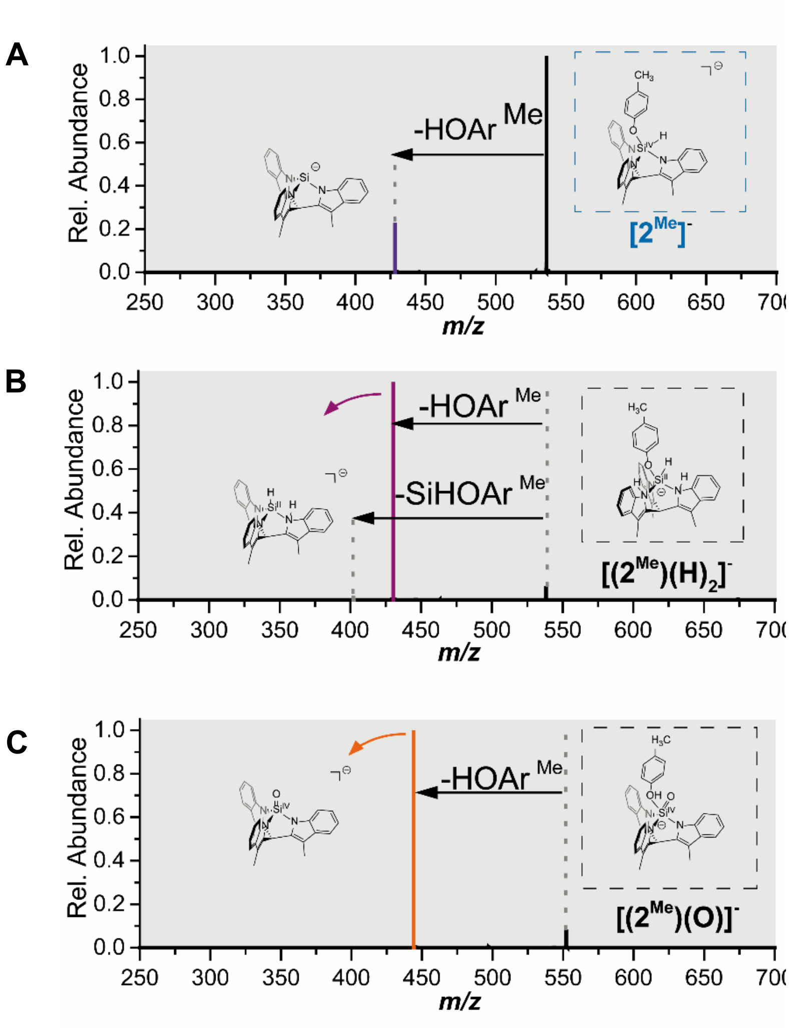


**Figure S3.** A) Collision-induced dissociation (CID) spectrum of mass-selected $\mathbf{[2^{Me}]^-}$ at collision voltage of 16 V. B) CID spectrum of mass-selected $\mathbf{[(2^{Me})(H)_2]^-}$ at the collision voltage of 16 V. C) CID spectrum of mass-selected $\mathbf{[(2^{Me})(O)]^-}$ at the collision voltage of 16 V.

In the case of **[(1)(OPh$^{Me}$)$_2$]$^-$**, since there is no favourable path of ligand dissociation or reductive elimination, the fragmentation at the same collision voltage is very small, and only fragments coming from complex decomposition are observed (Figure S4, A). On the contrary, **[(1)(HOPh$^{Me}$)$_2$]$^-$**, where phenol molecules are present as weakly bound ligands, shows almost full fragmentation for the loss of one phenol molecule (Figure S4, B), corroborating the proposal that for the decomposition compounds, the easy loss of the phenol molecule comes from ligand decoordination while for **2$^{Me}$** the phenol loss comes from a reductive elimination step.

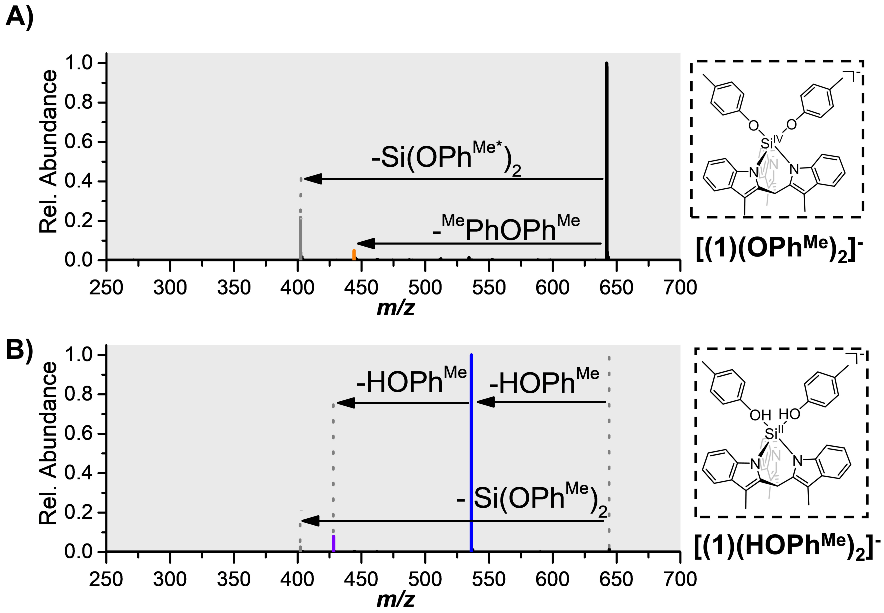


**4.** A) Collision-induced dissociation (CID) spectrum of mass-selected **[(1)(OPh$^{Me}$)$_2$]$^-$** at the collision voltage of 16 V. *Corresponds to [OPh$^{Me}$ – H]. B) CID spectrum of mass-selected **[(1)(HOPh$^{Me}$)$_2$]$^-$** at the collision voltage of 16 V.

### 2.3. Electronic modification of $2^R$ to modulate the reductive elimination step

Once we were sure we can detect the oxidative addition product $\mathbf{2^{Me}}$ and promote its reductive elimination in the gas phase, we wanted to explore how electronic modifications on the phenol molecule can affect the reductive elimination step. To explore them, and due to the high sensitivity of these samples, the oxidative addition products were generated in situ by mixing a 0.1 mM solution of precursor **1** in dry acetonitrile, prepared inside the glovebox, with different para-substituted phenols and -40°C, and rapidly injected to the mass spectrometer, under an argon overpressure. An immediate peak appeared corresponding to the desired oxidation product $\mathbf{2^R}$ (R = OMe, H, Cl, CN) (Figure S5). Collision-induced dissociations of mass-selected $\mathbf{2^R}$ showed the loss of $\mathbf{HOPh^R}$ as the exclusive fragmentation pathway. Energy-resolved collision-induced dissociation experiments were performed for all $\mathbf{2^R}$. Due to the sensitivity of the compounds used, we must have performed the experiments with the cryospray-QTOF instrument (timsTOF). We could not extrapolate the experiments to the single-collision conditions due to the small signal. Therefore, we couldn't do the kinetic simulation to extract quantitative bond dissociation energies. Instead, we present our results only as a qualitative measure. We report $E_{50\%}$ values (the energy where the ion intensities of the precursor and fragment ions are equal). Plotting the $E_{50\%}$ energy values of different $\mathbf{2^R}$ against their corresponding Hammett parameter shows a linear tendency where electron-withdrawing groups require less energy to promote the reductive elimination step (Figure 3C). This behaviour unveils how acidity modulates the reductive elimination step. More acidic phenols facilitate reductive elimination.

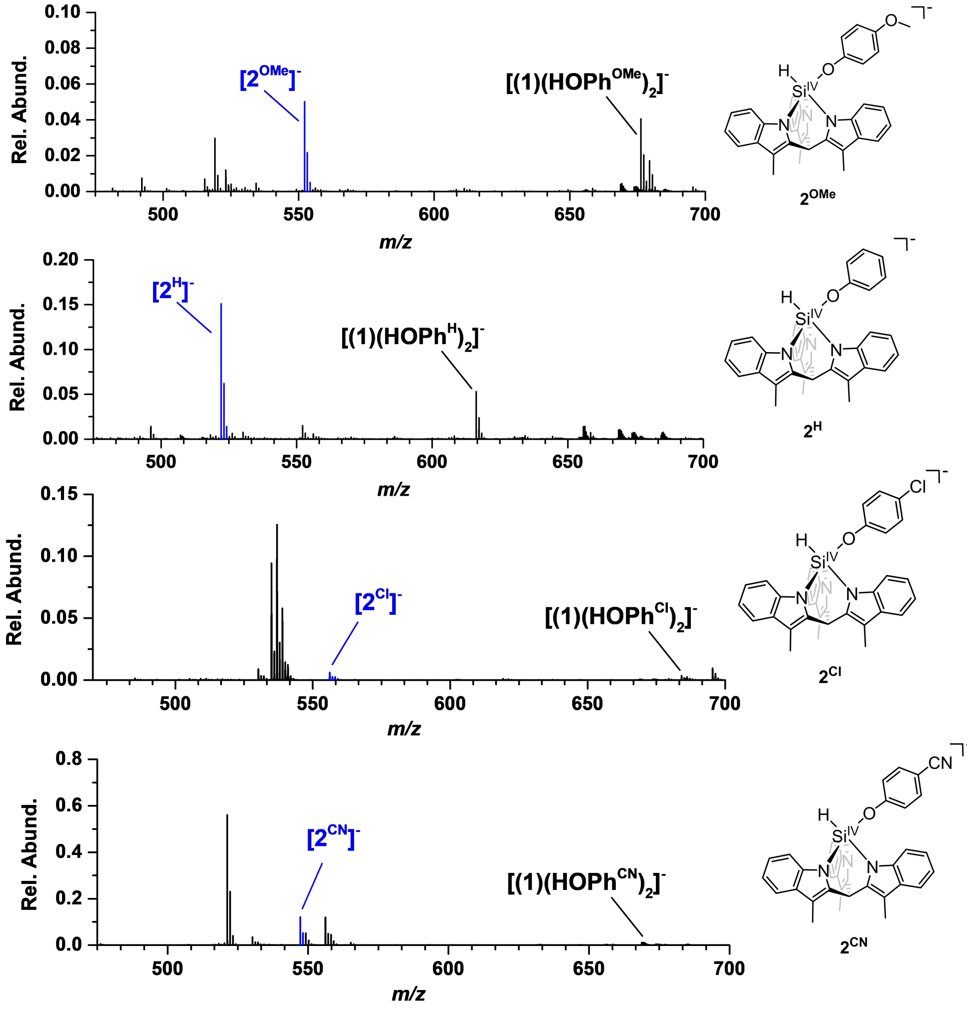


**Figure S5.** ESI-MS spectra of $[\mathbf{2^R}]^-$ bearing different para-substituted phenols.

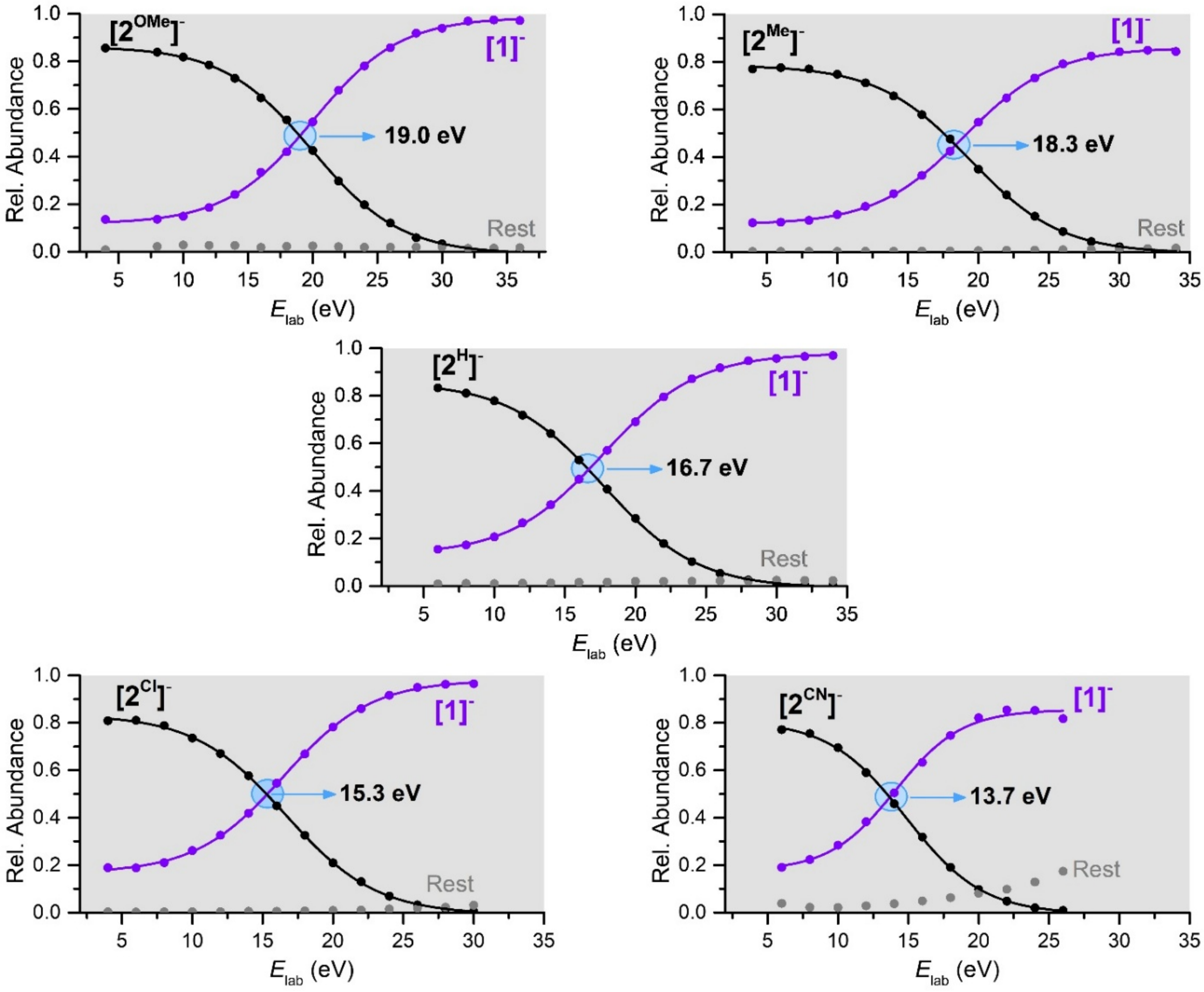


**Figure S6.** Energy-resolved CID curves for the reductive elimination of **[2$^{R}$]$^{-}$** to **[1]**.

**Losses observed at "Rest" in figure S7**

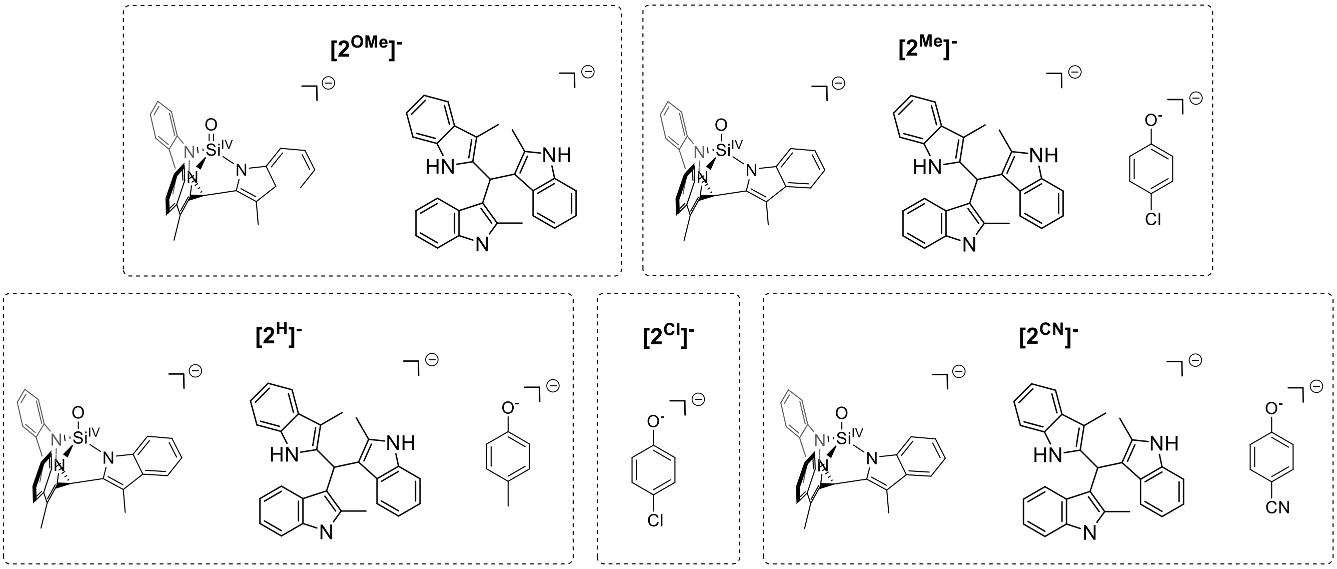


**Figure S7.** Additional fragments loss during CID of [2$^{R}$], labelled as rest in Figure S7.

### 2.4. ESI-MS of 3

Due to the complexity of the mass spectrum of the desired **[3]**$^{-}$ ion (*m/z* = 535.21), its mass-selection also included isobaric impurities from ions *m/z* = 535.18 (*') and *m/z* = 535.33 (**'), identified as the first $^{13}$C isotopes of unknown ions (*) and (**) (see Figure S8). Collision-induced dissociation at the same collision voltage of *m/z* = 535 and *m/z* = 534 allowed us to identify which fragments correspond to the fragmentation of the isobaric impurities (labelled in red in Figure S9) and neglect them.

The energy-resolved CID curves for the reductive elimination of **[3]**$^{-}$ to **[1]**$^{-}$ in Figure 4 were constructed by considering only the blue ions in Figure S10, corresponding to fragment from **[3]**, while ignoring red-labelled ions, which represent unidentified impurities. The fragments of these impurities could not be identified, with the primary ions detected at *m/z* = 444.15, *m/z* = 358.21 and *m/z* = 163.11.

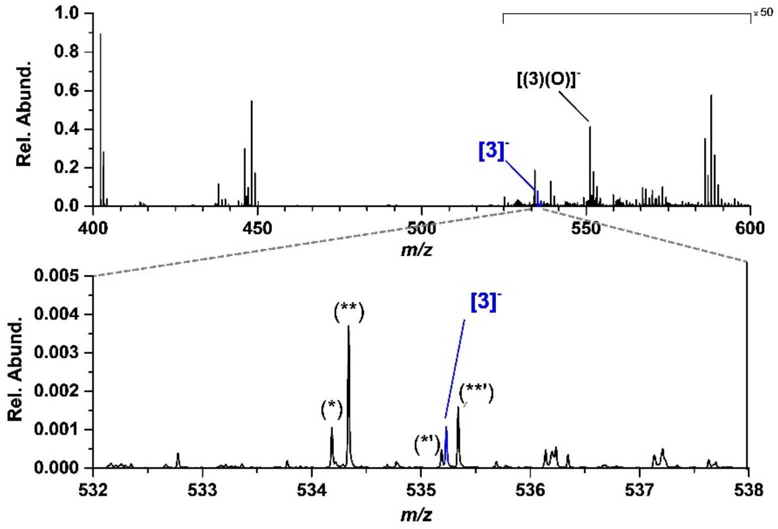


**Figure S8.** ESI-MS spectra of **[3]**$^{-}$.The desired **[3]**$^{-}$ ions with *m/z* = 535.21 were co-mass selected with isotopic impurities from unknown ions having *m/z* = 535.18 and *m/z* = 535.33. Collision-induced dissociation at the same collision energy of *m/z* = 535 and *m/z* = 534 allowed us to identify which fragments correspond to the fragmentation of the isobaric impurities and neglect them in the energy-resolved CID of **[3]**$^{-}$.

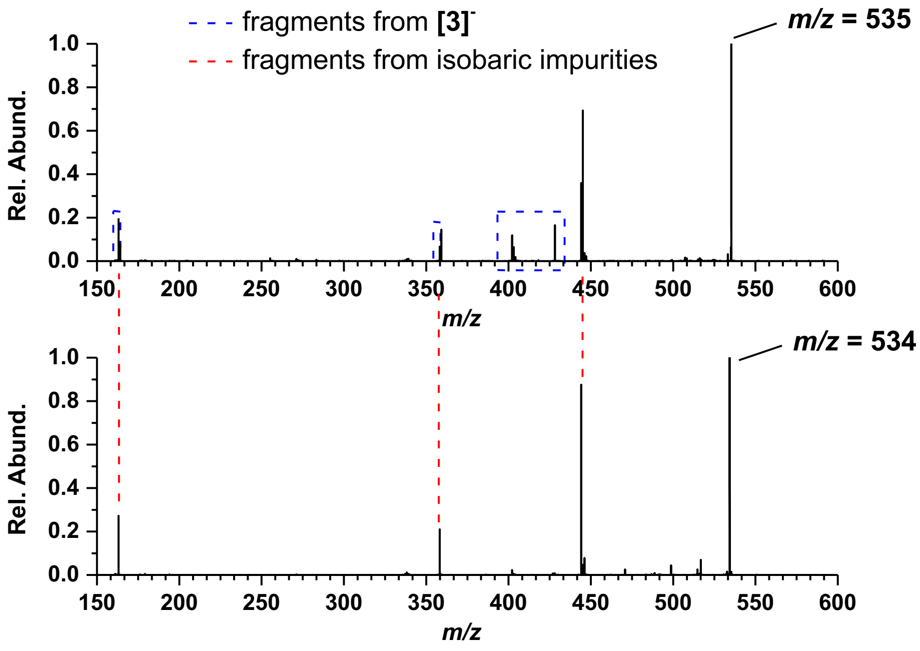


**Figure S9.** Collision-induced dissociation spectra of *m/z* = 535, **[3]**$^{-}$ (top) and *m/z* = 534 (bottom) at collision voltage 32 V to discern between fragments of the desired ion (blue dashed line) and fragments coming from impurities (red dashed line).

## 3. DFT studies

### 3.1. Computational methods

All calculations were performed using Gaussian 16, Revision C.01[3] Geometry optimizations were carried out in vacuum at the B3LYP-GD3BJ/6-31G(d,p) level of theory. Single point calculations were carried at a higher level of theory at B3LYP-GD3BJ/6-311++G(d,p). Frequency analyses on all stationary points were used to ensure that they are minima (no imaginary frequency) or transition states (one imaginary frequency). Relaxed potential energy surface (PES) scans were carried out in vacuum at B3LYP-GD3BJ/6-31G(d,p) level of theory. Related stationary points were optimized at the same level of theory. Coordinates along with the corresponding energies are provided in Section 4.

### 3.2. Study of the RE mechanism

Different mechanisms were considered and studied for the RE process which are based on previous data[4] (scheme S1). The initial formation of the corresponding Lewis acid−base complex (Si-O–H) followed by the loss of the phenol (a) or a concerted process (c) were considered but discarded since int(A) could not be optimized and the transition state (B) could be located. Instead, the DFT data suggests a rebound-like ionic mechanism, which is the microscopic reverse of that computed for the oxidative addition of phenols to cage silanides in solution. First, the $ArO^-$ anion group detaches from the silicon center to form a loosely bound ion-molecule complex (**int1**). Then, the phenolate abstracts a proton from the neutral silane to form a hydrogen-bonded phenol/silanide complex (**int2**), from which the phenol is released as a neutral fragment (Scheme S2).

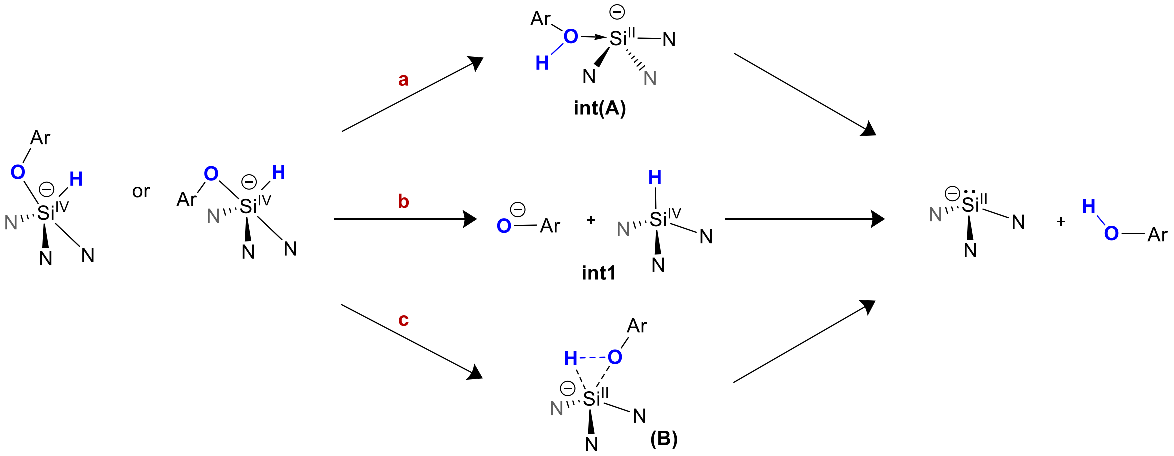


**Scheme S1.** Hypothetic mechanisms for the reductive elimination **2**.

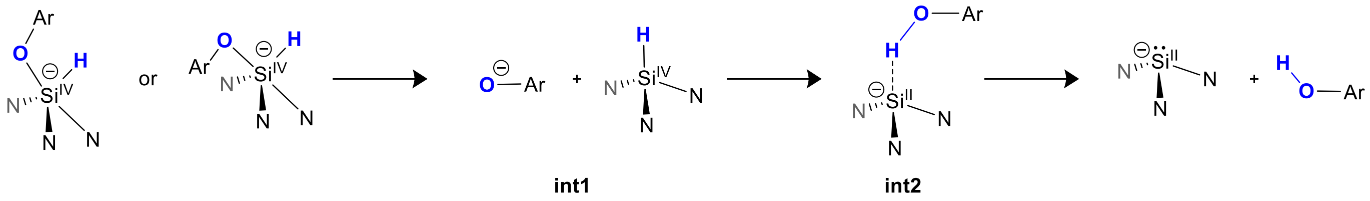


**Scheme S2.** Proposed ionic mechanism for the reductive elimination.

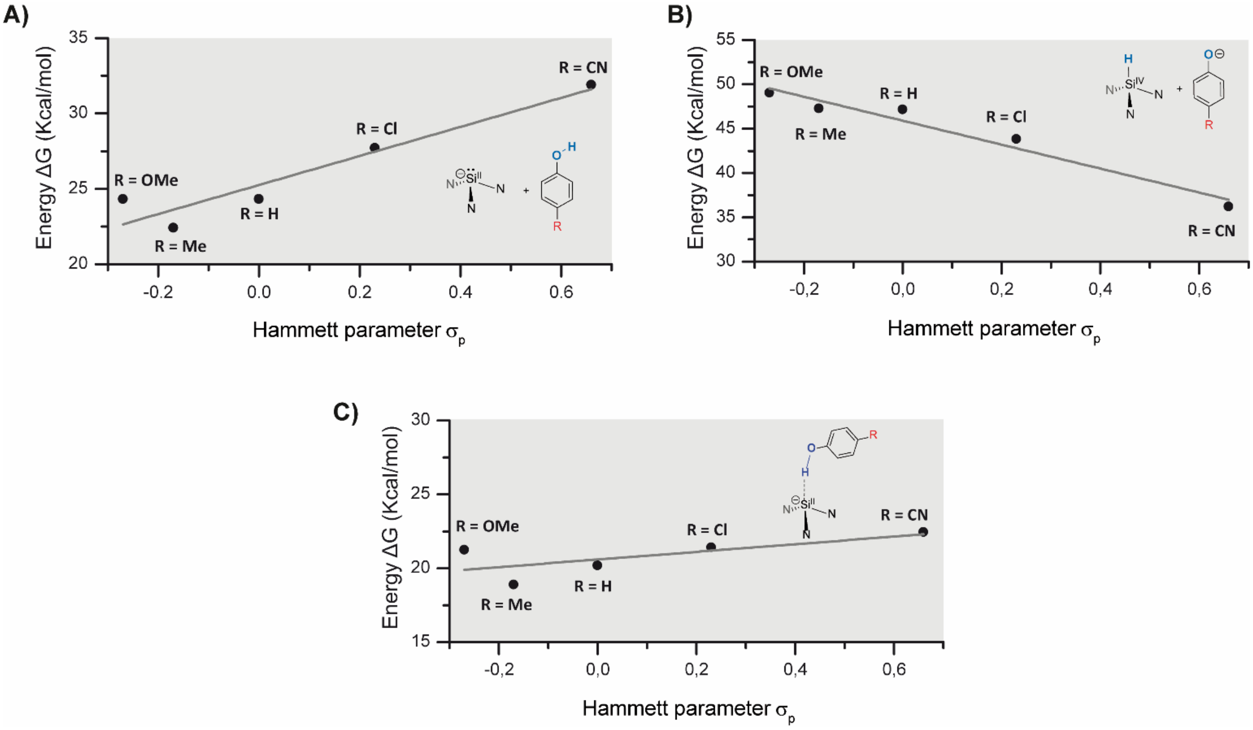


**Scheme S3.** Hammett plot representing the energy of the products (A), int1 (B) and int2 (C) for the different substituted phenols vs the Hammett parameter for substituents in para position

In addition, a more complex mechanism involving initial cleavage of an Si–N bond instead of the strong Si–O bond was computed for **2^Me^** (Figure S10). The mechanism involves first a hydrogen shift from Si to one of the N atoms of the cage leading, via TS1, to a structure in which the the cage is open (**NH transfer open**). A pyramidal inversion at Si (**$TS_{inv}$**) is required to bring the Si–O bond in proximity to the N–H bond (**NH transfer close**). From here, the Ar-O$^-$ fragment detaches from the Si center, leaving behind a 2-coordinate silylene fragment, to form a hydrogen bond wiht the indole N–H moiety (**Int O-H-N**). Then, the H is trasferred to the Ar-O$^-$ fragment (**Int N-H-O**) via **TS2**. Finally, cresol dissociation must happen (**Int open cage**) before cage closure to the final products, as supported by the relaxed PES scan for N–H elongation from **Int N-H-O** (Figure S11). For this reason, the energy of **Int open cage** was estimated from a constrained geometry optimization in which the longest Si–N distance was frozen, and then calculating its frequency to determine the thermal correction and a single point calculation to get the its final energy. Because both the calculated barrier for pyramidal inversion at Si (57.7 kcal/mol) and the estimated barrier for dissociation (63.7 kcal/mol) are significantly higher than that of the proposed ionic mechanism (47.4 kcal/mol), this H-shift mechanism can be ruled out.

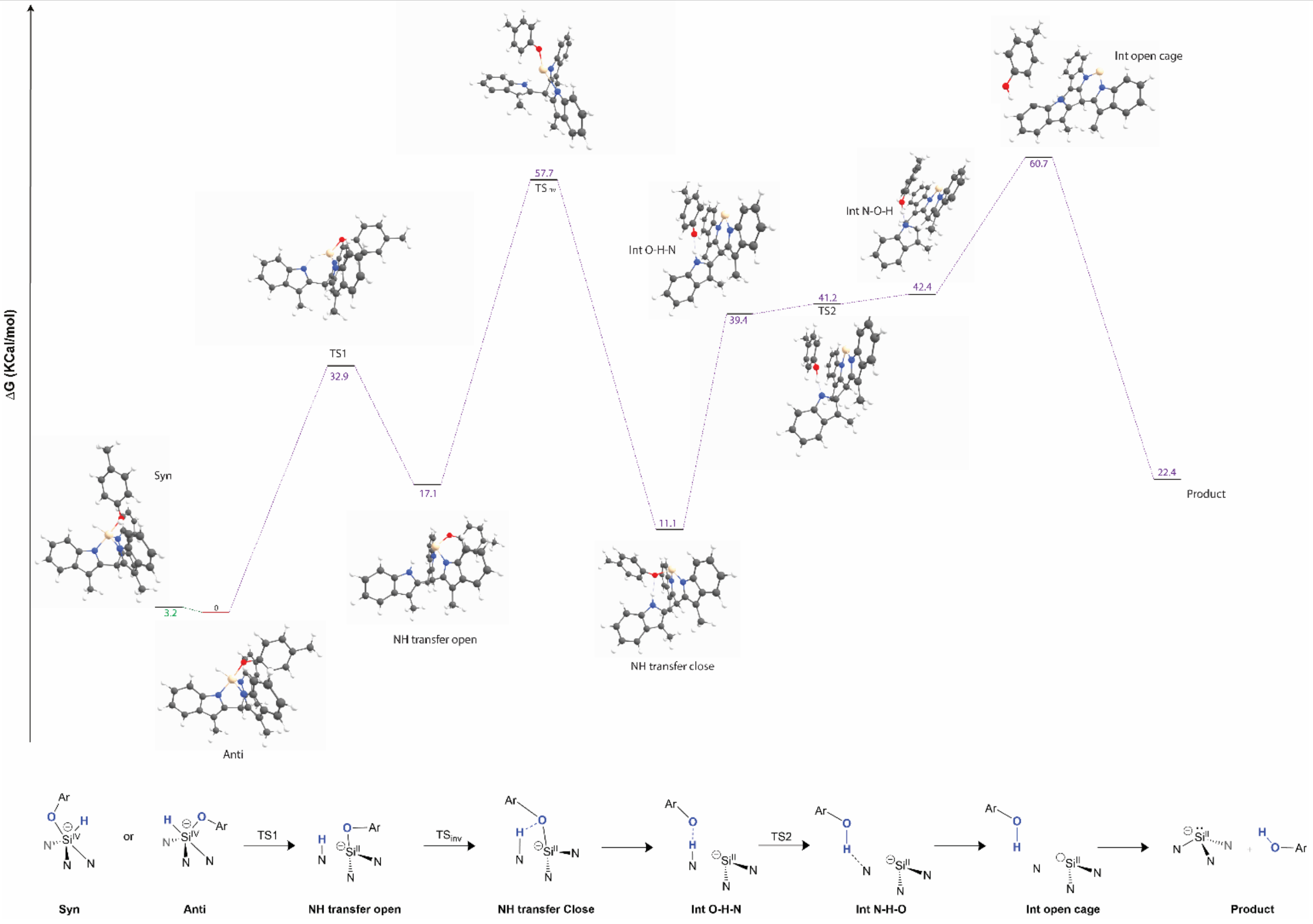


**Figure S10.** Computed free energies for the hypothetic H-shift mechanism for reductive elimination from **2$^{Me}$** at the B3LYP-GD3BJ/6-311++G(d,p)//6-31G(d,p) level of theory (Top) and scheme of the reaction mechanism (bottom). The open structure of the tmimSi⁻ anion in **Int open cage** was optimized with the longest Si–N distance constrained to its value in the last frame of the relaxed PES scan represented in Figure S11.

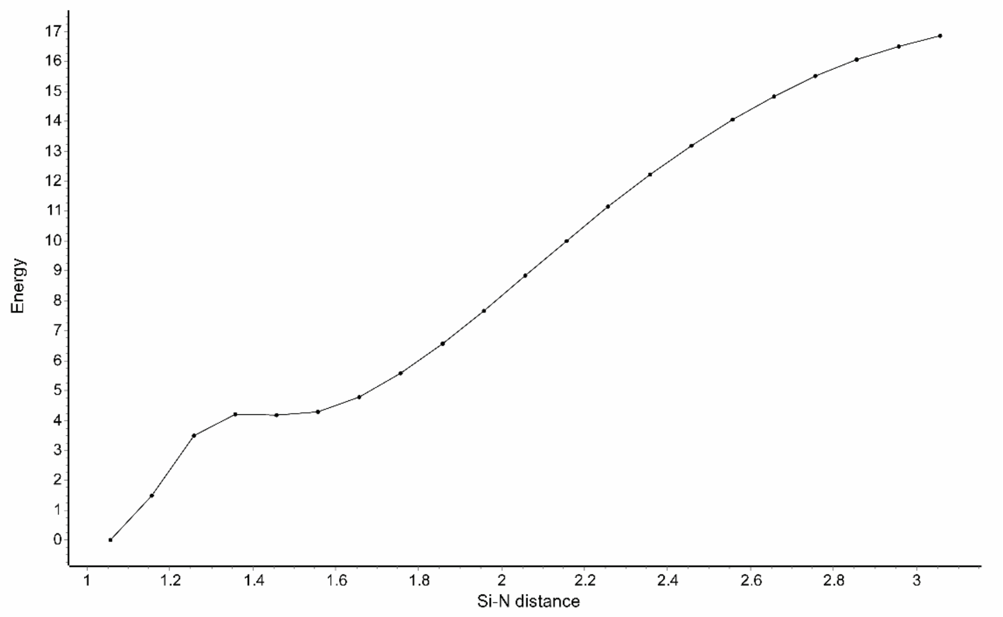


**Figure S11.** Relaxed PES scan for N–H elongation from **Int N-H-O**.

## 3.3. Energies

Table **S1**. Electronic energies (B3LYP/6-311++G(d,p)//6-31G(d,p)) and Thermal corrections (B3LYP/6-31G(d,p)) for all structures. Cartesian coordinates are provided in a separate .xyz file.

| Compound | SP Energy (Hartree) | Thermal correction (Hartree) |
|---|---|---|
| **4-hydroxybenzonitrile** | | |
| ***tmimSi*** | -1 534.62605782 | 0.372617 |
| ***CNPhO-*** | -399.31644664 | 0.058667 |
| ***tmimSiHOPhCN*** | -1 934.54166519 | 0.465902 |
| ***tmimSiH*** | -1 535.1519039 | 0.383106 |
| ***PhCNOH*** | -399.851942938 | 0.07193 |
| ***tmimSi----CNPhOH*** | -1 934.5114422 | 0.462973 |
| ***tmimSiCNPhOH anti*** | -1 934.5506759 | 0.466435 |
| **p-cresol** | | |
| ***tmimSi*** | -1 534.62605782 | 0.372617 |
| ***CH3PhO-*** | -346.343738457 | 0.086264 |
| ***tmimSiHOPhCH3*** | -1 881.59006116 | 0.493662 |
| ***tmimSiH*** | -1 535.1519039 | 0.383106 |
| ***PhCH3OH*** | -346.91168459 | 0.099244 |
| ***tmimSi----CH3PhOH*** | -1 881.5612629 | 0.489772 |
| ***tmimSiCH3PhOH anti*** | -1 881.5974967 | 0.495921 |
| ***NH tranfer close*** | -1.881.57569165 | 0.491851 |
| ***TS1*** | -1881.53504302 | 0.485886 |
| ***NH transfer open*** | -1.881.56819711 | 0.49381 |
| ***Int N-H-O*** | -1.881.53532313 | 0.496499 |
| ***TS2*** | -1.881.52846674 | 0.492532 |
| ***Open cage (freezed)*** | -1.534.55923475 | 0.366775 |
| ***$TS_{inv}$*** | -1.881.50083199 | 0.491209 |
| ***Int O-H-N*** | -1.881.53088542 | 0.496921 |
| **p-chlorophenol** | | |
| ***tmimSi*** | -1 534.62605782 | 0.372617 |
| ***ClPhO-*** | -766.65035222 | 0.050213 |
| ***tmimSiHOPhCl*** | -2 301.88885168 | 0.457881 |
| ***tmimSiH*** | -1 535.1519039 | 0.383106 |
| ***PhClOH*** | -767.205196065 | 0.06407 |
| ***tmimSi----ClPhOH*** | -2 301.8594102 | 0.45476 |
| ***tmimSiClPhOH anti*** | -2 301.8971139 | 0.458366 |
| **Phenol** | | |
| ***tmimSi*** | -1 534.62605782 | 0.372617 |
| ***PhO-*** | -307.01382212 | 0.061866 |
| ***tmimSiPhOH*** | -1 842.25868366 | 0.469705 |
| ***tmimSiH*** | -1 535.1519039 | 0.383106 |
| ***PhOH*** | -307.579615273 | 0.075893 |
| ***tmimSi----PhOH*** | -1 842.2296380 | 0.465916 |
| ***tmimSiPhOH anti*** | -1 842.2658955 | 0.470021 |

| p-methoxyphenol | | |
|---|---|---|
| ***tmimSi*** | -1 534.62605782 | 0.372617 |
| ***OCH3PhO-*** | -421.57048622 | 0.090286 |
| ***tmimSiHOPhOCH3*** | -1 956.81865502 | 0.498735 |
| ***tmimSiH*** | -1 535.1519039 | 0.383106 |
| ***PhOCH3OH*** | -422.139868664 | 0.104876 |
| ***tmimSi----OCH3PhOH*** | -1 956.7906300 | 0.497323 |
| ***tmimSiOCH3PhOH anti*** | -1 956.82454772 | 0.497398 |

## 4. References

[1] R. S. Ghadwal, H. W. Roesky, S. Merkel, J. Henn, D. Stalke, *Angewandte Chemie - International Edition* **2009**, *48*, 5683–5686.

[2] L. Witteman, T. Evers, M. Lutz, M. E. Moret, *Chemistry - A European Journal* **2018**, *24*, 12236–12240.

[3] M. J. Frisch, G. W. Trucks, H. B. Schlegel, G. E. Scuseria, M. A. Robb, J. R. Cheeseman, G. Scalmani, V. Barone, G. A. Petersson, H. Nakatsuji, X. Li, M. Caricato, A. V Marenich, J. Bloino, B. G. Janesko, R. Gomperts, B. Mennucci, H. P. Hratchian, J. V Ortiz, A. F. Izmaylov, J. L. Sonnenberg, Williams, F. Ding, F. Lipparini, F. Egidi, J. Goings, B. Peng, A. Petrone, T. Henderson, D. Ranasinghe, V. G. Zakrzewski, J. Gao, N. Rega, G. Zheng, W. Liang, M. Hada, M. Ehara, K. Toyota, R. Fukuda, J. Hasegawa, M. Ishida, T. Nakajima, Y. Honda, O. Kitao, H. Nakai, T. Vreven, K. Throssell, J. A. Montgomery Jr, J. E. Peralta, F. Ogliaro, M. J. Bearpark, J. J. Heyd, E. N. Brothers, K. N. Kudin, V. N. Staroverov, T. A. Keith, R. Kobayashi, J. Normand, K. Raghavachari, A. P. Rendell, J. C. Burant, S. S. Iyengar, J. Tomasi, M. Cossi, J. M. Millam, M. Klene, C. Adamo, R. Cammi, J. W. Ochterski, R. L. Martin, K. Morokuma, O. Farkas, J. B. Foresman, D. J. Fox, **2016**.

[4] P. A. Benzan Lantigua, M. Lutz, M. E. Moret, *Angewandte Chemie - International Edition* **2024**, *63*, DOI 10.1002/anie.202319899.

## Author Contributions

M.-E.M and J. R. initiated and supervised the project. P.A. B. L. synthesised the compounds, prepared the samples, and performed the DFT calculations. M. R. performed the ESI-MS experiments. M.R. and J. R. Interpreted the ESI-MS data. M.-E.M and P.A.B.L. interpreted the DFT calculations. P.A. B. L. and M.R. wrote the manuscript with input from M.-E.M. and J.R. All authors approved the final manuscript.
